# IL-1β Stimulates COX-2 Dependent PGE_2_ Synthesis and CGRP Release in Rat Trigeminal Ganglia Cells

**DOI:** 10.1371/journal.pone.0017360

**Published:** 2011-03-04

**Authors:** Lars Neeb, Peter Hellen, Carsten Boehnke, Jan Hoffmann, Sigrid Schuh-Hofer, Ulrich Dirnagl, Uwe Reuter

**Affiliations:** 1 Department of Neurology and Experimental Neurology, Charité Universitätsmedizin Berlin, Berlin, Germany; 2 Department of Neurology, Universitätsklinikum Tübingen, Tübingen, Germany; Charité-University Medicine Berlin, Germany

## Abstract

**Objective:**

Pro-inflammatory cytokines like Interleukin-1 beta (IL-1β) have been implicated in the pathophysiology of migraine and inflammatory pain. The trigeminal ganglion and calcitonin gene-related peptide (CGRP) are crucial components in the pathophysiology of primary headaches. 5-HT1B/D receptor agonists, which reduce CGRP release, and cyclooxygenase (COX) inhibitors can abort trigeminally mediated pain. However, the cellular source of COX and the interplay between COX and CGRP within the trigeminal ganglion have not been clearly identified.

**Methods and Results:**

1. We used primary cultured rat trigeminal ganglia cells to assess whether IL-1β can induce the expression of COX-2 and which cells express COX-2. Stimulation with IL-1β caused a dose and time dependent induction of COX-2 but not COX-1 mRNA. Immunohistochemistry revealed expression of COX-2 protein in neuronal and glial cells. 2. Functional significance was demonstrated by prostaglandin E2 (PGE_2_) release 4 hours after stimulation with IL-1β, which could be aborted by a selective COX-2 (parecoxib) and a non-selective COX-inhibitor (indomethacin). 3. Induction of CGRP release, indicating functional neuronal activation, was seen 1 hour after PGE_2_ and 24 hours after IL-1β stimulation. Immunohistochemistry showed trigeminal neurons as the source of CGRP. IL-1β induced CGRP release was blocked by parecoxib and indomethacin, but the 5-HT1B/D receptor agonist sumatriptan had no effect.

**Conclusion:**

We identified a COX-2 dependent pathway of cytokine induced CGRP release in trigeminal ganglia neurons that is not affected by 5-HT1B/D receptor activation. Activation of neuronal and glial cells in the trigeminal ganglion by IL-β leads to an elevated expression of COX-2 in these cells. Newly synthesized PGE_2_ (by COX-2) in turn activates trigeminal neurons to release CGRP. These findings support a glia-neuron interaction in the trigeminal ganglion and demonstrate a sequential link between COX-2 and CGRP. The results could help to explain the mechanism of action of COX-2 inhibitors in migraine.

## Introduction

Pro-inflammatory cytokines have been linked to inflammation and pain [1]. Interleukin-1β (IL-1β), interleukin-6 and tumor necrosis factor-α (TNFα) are known to induce hyperalgesia in rats [2]–[4]. Cytokines also seem to play an important role in pathophysiological mechanisms involved in migraine headache. Among others, IL-1β and TNFα levels were elevated in jugular vein blood during migraine attacks [5], [6]. Plasma levels of IL-6 were also increased in patients with migraine compared to healthy controls [7]. Furthermore, enhanced expression of IL-1β was found in the meninges in an experimental animal model related to migraine [8].

The trigeminal system, neuropeptides and inflammatory mediators are key players in the pathophysiology of migraine. Activation of perivascular trigeminal nerves within meninges causes the release of calcitonin gene-related peptide (CGRP) and other peptides e.g. substance P [9], [10]. This leads to a series of peripheral and central events such as vasodilatation, plasma protein extravasation [11] and neuronal activation [12].

CGRP is classified as the most important neuromediator in the pathophysiology of migraine and other primary headaches. It is believed not only to be involved in dilation of cerebral and dural blood vessels but also in release of inflammatory mediators from mast cells and transmission of nociceptive information [13]. In clinical studies, plasma levels of CGRP can be found to be elevated during migraine and cluster headache attacks [14], [15]. Intravenous injection of CGRP induces a typical headache in migraineurs [16] and CGRP receptor antagonists (BIBN4096BS/MK-0974) can abort attacks [17], [18].

On a cellular basis in an experimental cell culture model, stimulation of trigeminal ganglia neurons with potassium chloride, capsaicin or a cocktail of inflammatory mediators used to mimic neurogenic inflammation resulted in an elevated CGRP release in these cells. Stimulus induced CGRP release could be repressed by the 5-HT_1B/D_ agonist sumatriptan [19], which is used in acute migraine treatment, and furthermore by botulinum toxin type A [20] and topiramate [21], two substances proved to be effective in migraine prophylaxis. Stimulation with TNFα increased the synthesis and release of CGRP in trigeminal ganglia neurons [22] indicating a link between cytokines and CGRP release.

In addition to CGRP, Cyclooxygenases (COX) are important peripheral mediators of inflammation and pain. COX enzymes are involved in migraine pathomechanisms as non-selective [23] and selective COX-2 inhibitors [24], [25] can abort attacks. The constitutively expressed isoform COX-1 and the inducible enzyme COX-2 both synthesize prostaglandins [26] which are involved in neuronal sensitization phenomena induced by Interleukin 1β (IL-1β) [27]. However, the precise pathophysiological role of COX and its reaction product prostaglandin E_2_ (PGE_2_) in migraine remain unclear.

We investigated the expression of COX and its cellular sources in cultured trigeminal ganglia cells (TGC) upon stimulation with the cytokine IL-1β. We further assessed the effects of IL-1β on CGRP release in vitro. Based on the efficacy of COX- inhibitors to abort migraine we hypothesized that induced COX-2 expression leads to PGE_2_ production in TGC which may have an effect on CGRP release.

## Materials and Methods

### Animals

We used 3 days old male and female Sprague Dawley rats (Charles River, Sulzheim, Germany). All animals were kept under standard laboratory housing conditions with a 12-h light–dark cycle and with an adult female Sprague Dawley rat (Charles River, Sulzheim, Germany) with free access to food and water. For cell culture procedures 3 day old rats were anaesthetized with an isoflurane vaporizer (4%) and decapitated. All animal work was carried out in accordance with the European Communities Council Directive of 24 November 1986 (86/609/EEC) regarding the care and use of animals for experimental procedures. The sacrifice of the rats and extraction of their brains was reported to and approved by the Landesamt für Gesundheit und Soziales Berlin (LaGeSo; T0322/96).

### Cell culture

Trigeminal ganglia cell culture was established as previously described [28] with minor modifications. In brief, trigeminal ganglia were dissected from 3 day old male and female Sprague Dawley rats (Charles River, Sulzheim, Germany). The cells were incubated for 90 min at 37°C in 10 ml dissociation medium (modified eagles medium; Biochrom, Berlin, Germany; with 10% bovine serum, 10 mM HEPES, 44 mM glucose, 100 U penicillin + streptomycin, 2 mM glutamine, 100 IE insulin/l) containing collagenase/dispase (final concentration 100 µg/ml) (Boehringer Mannheim, Germany), rinsed twice with phosphate buffered saline (PBS) 0.1 M and again incubated with trypsin/EDTA (0.05%/0.02% w/v in PBS) for 30 min for dissociation. Subsequently, cells were rinsed twice with PBS and once with dissociation medium, dissociated by Pasteur pipette and pelleted by centrifugation at 2100× g for 2 min at 21°C. After suspension in starter medium (Invitrogen, Karlsruhe, Germany) plus 1% penicillin/streptomycin, 0,25% L-glutamine, 2% B27-supplement, 0,1% 25 mM glutamate, 2.5 mM calcium chloride and 100 ng/ml NGF-β, cells were plated in 24 well plates and filled to 500 µl with starter medium at a density of 0.5×10^−6^ cells/cm^2^ (equates approximately 2 ganglia/well). Cells used for immunohistochemistry were seeded on round glass cover slips previously inserted in the well plates. Wells were pretreated by incubation with poly-l-lysin (5% w/v in PBS) for 90 minutes at 4°C, then rinsed with PBS, followed by incubation with coating medium (dissociation medium with 1% w/v collagen G) for 90 minutes at 37°C in the incubator. After that, the wells were rinsed twice with PBS and filled with starter medium in which cells were seeded. Cytosine arabinoside (final concentration 10 µM; Sigma Aldrich, Munich, Germany) was added at day 1 and day 3 to minimize growth of non-neuronal cells. Cultures were kept at 37°C and 5% CO_2_ and fed with neurobasal medium + B27 medium every second day by replacing 50% of the medium. Condition of cultures was assessed by light microscopy, cell types by cell morphology and immunohistochemistry (with antibodies against glial fibrillary acidic protein (GFAP) for glial cells and β-tubulin III for neurons). Cell damage was monitored by life-death assays. Stimulation experiments and immunohistochemistry were performed on day 6.

### Quantitative real-time RT-PCR

Cultured trigeminal ganglia cells were stimulated with recombinant rat IL-1β (R&D Systems, Wiesbaden, Germany; 10 ng/ml, diluted in PBS 0.1 M) or vehicle (0.1 M PBS). In experiments with the IL-1 receptor antagonist (IL-1ra, R&D Systems, Wiesbaden, Germany) cells were preincubated with 10 µg/ml IL-1ra 15 min prior to stimulation with 10 ng/ml IL-1β. The supernatant was removed carefully with a pipette at certain time points (1.5, 3 and 6 hours). Total cellular RNA of two equally treated wells was extracted using Trizol reagent (Invitrogen, Karlsruhe, Germany) according to the supplier's manual. RNA preparation and cDNA synthesis were performed as described previously by our laboratory [29]. Real time reverse transcription quantitative polymerase chain reaction (RT-PCR) was carried out on a LightCycler (Roche Diagnostics GmbH, Mannheim, Germany) using the LC-Fast Start DNA Master SYBR Green I kit as recommended by the manufacturer and primers for CGRP, COX-1, COX-2 and GAPDH (Promega, Mannheim, German). The DNA polymerase was heat activated at 95°C followed by thermal cycling until saturation. Table 1 lists the used primer sequences, cycling conditions and primer efficacies. Specificity of all primers was confirmed by sequencing of amplicons. Each essay was run in duplicate. LightCycler Relative Quantification Software was used for amplification, detection and determination of the crossing point. The relative expression ratio for each time point of the measured genes was calculated from the previously determined RT-PCR efficiencies (E) of the primers and the crossing point deviation (ΔCP) of the sample (treated with IL-1β or vehicle) versus the control (0 hour time point) with normalization for RNA preparation and reverse transcriptase reaction on the basis of its mRNA content of the housekeeping gene Glycerinaldehyd-3-phosphat-Dehydrogenase [30].

**Table 1:** Sequences, cycling conditions and efficacies of all primers used in qRT-PCR.

Gene	Primer (5′-3′)	Den.	Ann.	Elo.	Efficacy
CGRP	forward AAG TTC TCC CCT TTC CTG GTreverse GAG ACC TTC AAC ACC CCA GCC	95°C15 s	66°C10 s	72°C15 s	1,918552
COX-1	forward TAA GTA CCA GGT GCT GGA TGGreverse GGT TTC CCC TAT AAG GAT GAG [59]	95°C15 s	58°C10 s	72°C 15 s	1,965760
COX-2	forward TGA TCG AAG ACT ACG TGC AAC ACreverse CAG CAA TCT GTC TGG TGA ATG AC	95°C15 s	63°C10 s	72°C10 s	1,702342
GAPDH	forward AGA TTG GCA ATG CAT GCreverse CCT TCT TGA TGT CAT CAT ACT TGG	95°C15 s	63°C10 s	72°C10 s	1,816696

### Western blot analysis

Trigeminal ganglia cells incubated 6 hours with IL-1β (10 ng/ml) or vehicle were homogenized with an electric grinder on ice in 250 µl buffer containing 10 mM HEPES (pH 7.4), 0.42 M KCl, 5 mM MgCl_2_, 1 mM EDTA, 1 mM EGTA, 1 mM PMSF, 1 mM DTT and a protease inhibitor cocktail (diluted 1∶50 in PBS; Sigma Aldrich, Munich, Germany). The specimen was centrifuged at 21200× g for 30 min at 4°C. Homogenization was performed as described previously [31]. Whole cell lysates were separated on tris-glycine gels (Invitrogen, Karlsruhe, Germany) and transferred onto a nitrocellulose membrane. Blots were incubated overnight with rabbit polyclonal anti COX-2 serum (1∶1250; 160126; Cayman Chemical, Ann Arbor, Michigan) and then probed with a goat anti-rabbit horseradish peroxidase coupled secondary antibody (1∶7500; Amersham, Little Chalfont, Buckinghamshire, UK). An enhanced chemoluminescence system (Luminol reagent, Santa Cruz, California) was used for visualization. For loading control, membranes were stripped and reprobed (2 hours) with a mouse polyclonal anti β-actin antibody (1∶5000; Sigma Aldrich, Munich, Germany) followed by 2 hours incubation with goat anti-mouse horseradish peroxidase coupled IgG (1∶7500; Amersham, Little Chalfont, Buckinghamshire, UK). For positive control macrophage + IFNy/LPS cell lysate was used as provided by the manufacturer (BDBiosciences, Heidelberg, Germany). Optical density measurement for COX-2 was performed by dividing the intensity of the COX-2 bands by the intensity of the house keeping protein (β-Actin, 1∶5000).

### Immunohistochemistry

Trigeminal ganglia cell cultures (day 6) were incubated 6 or 24 hours with 10 ng/ml IL-1β or vehicle, subsequently rinsed with PBS 0.1 M (pH 7.4) and fixed with methanol 100% for 15 min at −20° (n = 4). Cells were washed 3 times with PBS 0.1 M and blocked with normal donkey serum 10% and 0.3% Triton X in 0.1 M PBS for 2 hours at 4°C. For COX-2/CGRP and β-tubulin III/GFAP co-staining cells were incubated overnight at 4°C in rabbit anti-COX-2 serum (diluted 1∶300 in PBS; 160126; Cayman Chemical, Ann Arbor, Michigan) or in rabbit anti-CGRP serum (diluted 1∶200 in PBS; C8198; Sigma Aldrich, Munich, Germany) and in mouse anti-β-tubulin III serum (as a specific neuronal marker) (diluted 1∶600 in PBS; T5076; Sigma Aldrich, Munich, Germany) or mouse anti glial fibrillary acidic protein (GFAP) serum (as a glia marker) (diluted 1∶300 in PBS; MAB360; Chemicon) +3% normal donkey serum and 0.3% Triton X in 0.1 M PBS. The specimen was then washed 3×10 min in PBS and incubated for 90 min with Alexa Fluor 594 donkey anti-rabbit IgG (diluted 1∶600 in PBS; A-21207; Invitrogen, Karlsruhe, Germany) and Alexa Fluor 488 donkey anti-mouse IgG (diluted 1∶600 in PBS; A-21202; Invitrogen, Karlsruhe, Germany) +3% normal donkey serum and 0.3% Triton X in 0.1 M PBS at room temperature.

Prior to use, all secondary antibodies were tested for non-specific staining by omitting the primary antibodies in both IL-1β and vehicle-treated cell cultures. Non-specific staining was not observed with any of the secondary antibodies.

Cover slips were placed on slides, air-dried, mounted with a glycerol-containing mounting medium (Mowiol, Calbiochem, Bad Soden, Germany) and observed on a fluorescent microscope (DMRA2, Leica GmbH, Wetzlar, Germany). Fluorescent images were photographed by digital camera (DC300F, Leica GmbH, Wetzlar, Germany) and captured with Leica DC Twain 5.1.10 (Leica GmbH, Wetzlar, Germany). Images were processed with Photoshop 6.0 (Adobe, San Jose, CA, USA) to visualize co-labelling by superimposing the digital images. Immunohistochemistry results of cell cultures were identical on day 2 and 6.

For co-staining of CGRP with COX-2 we used an Alexa Fluor 488 donkey anti-mouse IgG (A-21202; Invitrogen, Karlsruhe, Germany) labeled rabbit anti-CGRP serum (diluted 1∶200 in PBS; C8198; Sigma Aldrich, Munich, Germany) and a rabbit anti-COX-2 serum (diluted 1∶300 in PBS) +3% normal donkey serum and 0.3% Triton X in 0.1 M PBS. For labeling of the anti-CGRP antibody we used the ApexTM Alexa Fluor 488 Antibody labeling Kit according to the instructions of the manufacturer. Labeling was performed to avoid cross binding with the use of the same anti-CGRP and anti-COX-2 antibodies that were utilized in the β-tubulin III/GFAP co-stainings before. Cells were incubated overnight at 4°C and COX-2 antibodies were recognized by Alexa Fluor 594 donkey anti-rabbit IgG (diluted 1∶600 in PBS). All other procedures (washing, incubation and mounting) were performed as described above. Cover slips on slices were observed on a fluorescent confocal microscope (Leica TCS SPE confocal microscope; Leica Microsystems, Wetzlar, Germany). Images were photographed by the system built in digital camera and captured with Leica Confocal Software (Leica, Wetzlar, Germany). Images were processed with ImageJ (National Institutes of Health) to visualize co-labelling.

### PGE_2_ determination by enzyme immunoassay

For PGE_2_ determination 6 day old cultured trigeminal ganglia neurons were incubated with 10 ng/ml IL-1β or equal volume of vehicle (PBS 0.1 M) for 30 min or 4 hours. For inhibition studies cell cultures were preincubated for 15 min with sumatriptan (10 µM), indomethacin (10 µM) or parecoxib (10 µM or 1 µM) prior to stimulation with IL-1β (10 ng/ml). In control experiments, equal volumes of vehicle (PBS 0.1 M) were added at the corresponding time. Prior to stimulation 50 µl supernatant of each well were removed to assess baseline content of PGE_2_. 30 min or 4 hours after stimulation the supernatants of two dishes were pooled and 100 µl of the supernatant were removed for PGE_2_ determination. PGE_2_ release was determined using a specific PGE_2_ enzyme immunoassay (Cayman Chemical, Ann Arbor, Michigan, USA) according to the manufacturer's instructions. The baseline samples of the two corresponding wells were also pooled and PGE_2_ content was determined. All samples were measured in duplicates. PGE_2_ release was determined in pg/ml as absolute increase over baseline values in the corresponding two wells.

### CGRP determination by enzyme immunoassay

After 6 days in culture the medium was gently removed and replaced with fresh medium without NGF to exclude effects of NGF on protein release. 1 hour later cells were stimulated with IL-1β (10 ng/ml), PGE_2_ (100 nm or 10 µm) or equal volume of vehicle (PBS 0.1 M). For inhibition studies cells were preincubated 45 min before stimulation with sumatriptan (10 µM or 100 µM), indomethacin (10 µM) or parecoxib (10 µM). Prior to stimulation 50 µl supernatant of each well were removed to assess baseline content of CGRP. After 1, 4, 10 or 24 hours the supernatants of two dishes were pooled and 100 µl were removed for CGRP determination using a specific CGRP enzyme immunoassay (SPIbio, Montigny le Bretonneux, France) as recommended by the manufacturer. For each experiment, one set of wells was treated with 60 mM KCl to determine the responsiveness of the cultures to depolarizing stimuli as described previously [19]. Cultures that exhibited a response less than 2-fold on CGRP release after the depolarizing stimulus were not analyzed. CGRP release was determined in pg/ml as absolute increase over baseline values in the corresponding two wells. All samples were measured in duplicates.

### Statistical analysis

For PCR statistical analysis was performed using variance analysis followed by Bonferroni correction. For PGE_2_ and CGRP studies values were first tested for normal distribution (Kolmogorov-Smirnov test) followed by an unpaired t-test to detect statistically significant differences between two groups using SPSS 17 statistical software (SPSS, Chicago, IL, USA). Statistical significance was assumed when p<0.05. Data are shown as mean ± standard error of the mean (SEM).

## Results

### Characterization of trigeminal ganglia cell culture

Trigeminal ganglia are a heterogeneous tissue containing neuronal cells, satellite cells and Schwann cells. Neurons were identified by their typical pseudo-unipolar morphology of sensory neurons and by staining with the neuronal marker β-tubulin III. Under our conditions the cell culture obtained from rat trigeminal ganglia contained approximately 10% β-tubulin III positive neurons. The rest of the cell population consisted of astrocytes staining positive for GFAP. Most of the sensory neurons were surrounded by GFAP positive glial cells (satellite glial cells).

### IL-1β induces COX-2 mRNA

**Figure 1:**
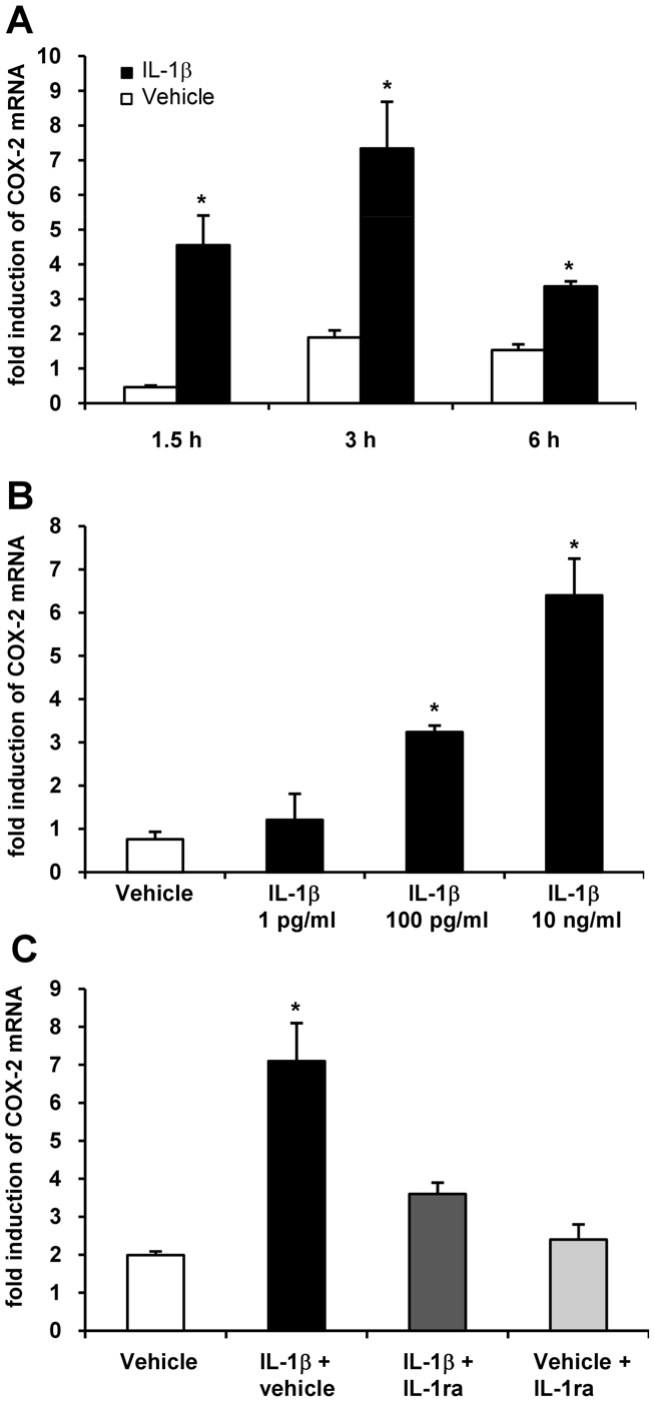
Expression of COX-2 mRNA in trigeminal ganglia cell culture following IL-1β incubation. Induction of COX-2 mRNA was time (**A**) and dose dependent (at the 3 hours time point; **B**). COX-2 mRNA expression was maximal after 3 hours (**A**) and at the IL-1β dose of 10 ng/ml (**B**) (n = 4-5/group). Co-incubation with the IL-1ra led to a significantly reduced COX-2 mRNA induction rate after 3 hours indicating the specificity of the effect (**C**). Application of the IL-1ra + vehicle resulted in a minor COX-2 mRNA induction rate comparable to vehicle administration (n = 3). Values are expressed as mean ± SEM. * p<0.05 compared to vehicle.

Incubation of cultured trigeminal ganglia cells with IL-1β (10 ng/ml) led to a time-dependent expression of COX-2 mRNA (Fig. 1A). COX-2 mRNA was significantly (∼4.5 fold) increased 90 min after incubation with IL-1β compared to vehicle (n = 4; p<0.05). COX-2 mRNA expression peaked after 3 hours (∼7 fold increase; n = 5; p<0.05) and declined after 6 hours but was still significantly (∼3 fold; n = 4; p<0.05) increased compared to vehicle stimulation.

To determine whether IL-1β induces COX-2 mRNA specifically, COX-1 mRNA expression was analyzed at the 3 hours time point. There was no difference between COX-1 mRNA expression in IL-1β (10 ng/ml) (0.49±0.02 SEM fold increase; n = 3) and vehicle stimulated cells (0.34±0.01 SEM fold increase; n = 3; p>0.05).

A dose response for IL-1β (1 pg/ml; 100 pg/ml; 10 ng/ml; 100 ng/ml) induced COX-2 gene expression was established at 3 hours since COX-2 mRNA expression was maximal at this time point. Increasing doses of IL-1β resulted in increased COX-2 mRNA levels (Fig. 1B). Stimulation with 1 pg/ml IL-1β showed no difference compared to stimulation with vehicle (n = 4; p>0.05). Higher doses of IL-1β (100 pg/ml; 10 ng/ml) led to a significant increase of COX-2 mRNA expression. Further increase of IL-1β doses (100 ng/ml) resulted in no further induction of COX-2 mRNA (data not shown). As IL-1β 10 ng/ml caused a reliable and strong COX-2 mRNA expression we used this dose for all further experiments.

To confirm the specificity of IL-1β induced COX-2 mRNA the IL-1 receptor antagonist (IL-1ra) (1 µg/ml) was added 15 min prior to IL-1β (10 ng/ml) to the supernatant. IL-1ra significantly reduced COX-2 expression rate: IL-1β plus vehicle resulted in a 7-fold COX-2 mRNA increase after 3 hours whereas co-administration with IL-1ra led to a 3.5-fold increase (n = 4; p<0.05) (Fig. 1C). Stimulation with IL-1β + IL-1ra was not significantly different from vehicle + IL-1ra administration alone (p>0.05).

### IL-1β induces COX-2 protein synthesis in neuronal and glial cells

**Figure 2:**
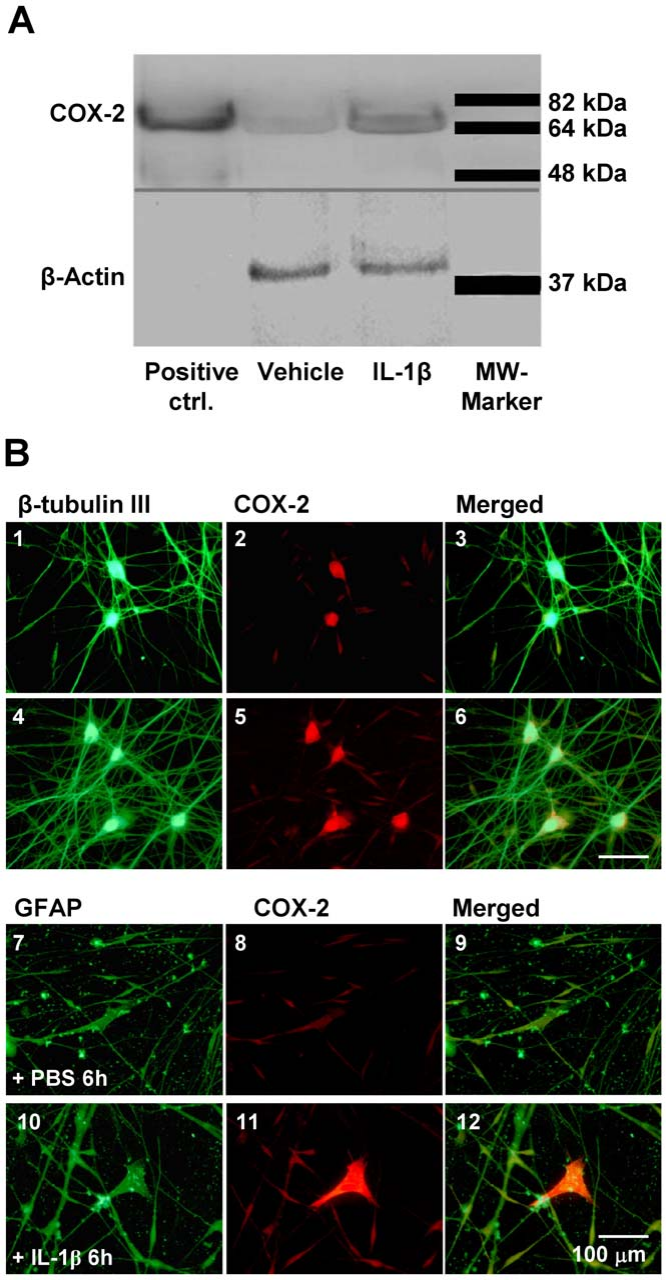
Expression of COX-2 protein in trigeminal ganglia cells after IL-1β stimulation. COX-2 protein in cell culture homogenates was analyzed 6 hours after stimulation with IL-1β using Western blot (n = 3). A representative image is shown in panel **A**. Cell lysate of IL-1β treated TGC and the positive control (IFNy/LPS treated macrophages) showed a clear band at 70 kDa corresponding to COX-2 protein. Vehicle stimulation resulted in a faint COX-2 expression. The expression of COX-2 protein in cultured trigeminal ganglia cells exposed 6 hours to vehicle (10 ng/ml, upper panel B1-B3/B7-B9) or IL-1β (0.1 M PBS, lower panel B4-B9/B10-B12) is shown in fluorescent micrographs in panel **B**. Cells were stained with a mouse β-tubulin III antibody, indicative of neuronal cells (B1 and B4) or a mouse GFAP antibody, indicative of glial cells (B7 and B10), and a rabbit COX-2 antibody (B2, B5, B8, B11). The β-tubulin III and the GFAP antibodies were recognized by an Alexa Fluor 488 labeled secondary donkey anti-mouse antibody (green) and the COX-2 antibody was recognized by an Alexa Fluor 594 labeled secondary donkey anti-rabbit antibody (red). Double stained cells appear orange in B3, B6, B9 and B12. IL-1β caused a clear upregulation of COX-2 in neuronal and glial cells (lower panel) whereas a faint COX-2 expression could also be observed in control experiments (upper panel). The strongest induction of COX-2 was seen in bigger glial cells (40-100 µm) and neuronal cells.

To show that enhanced COX-2 transcription leads to increased protein synthesis western blot analysis was performed after 6 hours. Immunoblot analysis of IL-1β stimulated trigeminal ganglia cell cultures (n = 3) and the cell lysate (positive control) revealed a single clear band after 6 hours at the size of approximately 70 kDA corresponding to COX-2 protein (Fig. 2A). In vehicle treated cultures (n = 3) a faint COX-2 band could be detected. However, there was a striking difference in signal intensity in all three experiments (optical density 0.15 ± 0.04 SD for vehicle vs. 0.48 ± 0.09 SD for IL-1β treated cells; p<0.05).

Induced COX-2 protein expression in trigeminal ganglia cell cultures could also be observed by immunohistochemistry with a COX-2 antibody 6 hours after treatment with IL-1β (10 ng/ml) (Fig. 2B; n = 4). Co-staining with mouse anti-β-tubulin III serum (β-tub III) for the identification of neuronal cells or with mouse anti-GFAP serum staining positive for glial cells revealed both neuronal and glial cells as the cellular source for COX-2 protein (Fig. 2B). Basal COX-2 expression could be observed in neuronal and glial cells and induction of COX-2 expression was seen also in both cell types. A strong induction of COX-2 was noted in particular in large glial cells.

### IL-1β induced PGE_2_ release is dependent on COX-2 activity

**Figure 3:**
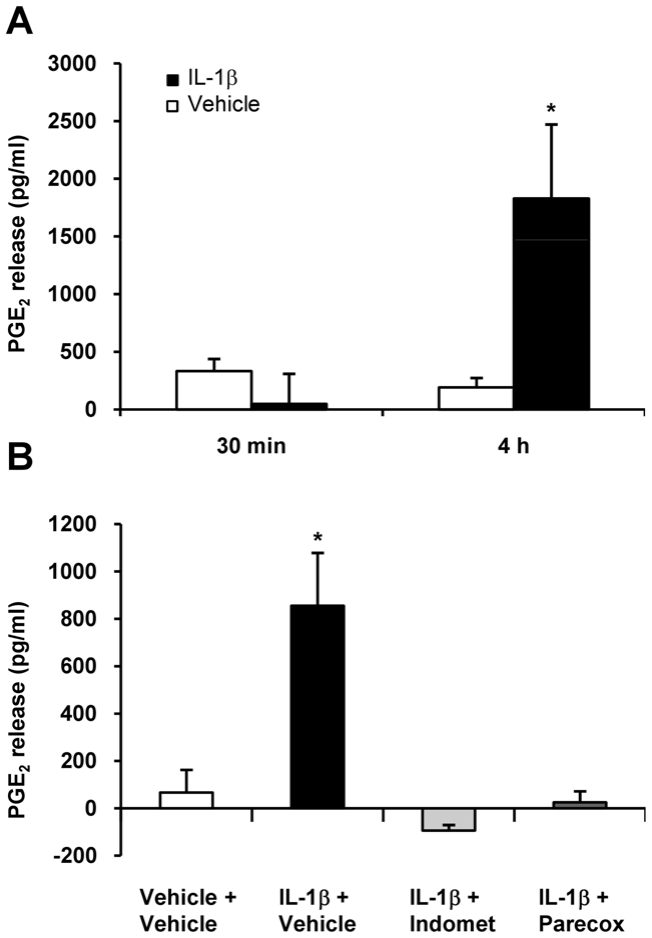
Induction of PGE_2_ levels in trigeminal ganglia cell culture after stimulation with IL-1β. PGE_2_ levels were significantly increased in TGC 4 hours after stimulation with IL-1β (10 ng/ml) compared to vehicle (**A**). No effect on PGE_2_ release was seen 30 minutes after stimulation with IL-1β compared to vehicle (n = 4/group). Dependency of IL-1β induced PGE_2_ release on COX-2 activity is shown in (**B**). 15 min of incubation with the selective (parecoxib, 10 µM) or the non-selective (indomethacin, 10 µM) COX-2 inhibitor completely aborted IL-1β induced PGE_2_ release after 4 hours. PGE_2_ levels are illustrated as mean pg/ml ± SEM compared to baseline. *p<0.05 compared to vehicle (n = 3-4/group).

To assess whether IL-1β induced COX-2 expression is functionally significant, PGE_2_ release into the supernatant was determined by Enzyme immunoassay (EIA). PGE_2_ release was measured before and after maximal induction of COX-2 mRNA (3 hours after stimulation with IL-1β). PGE_2_ content in the supernatant was not significantly different 30 mins after stimulation with IL-1β (48±261 SEM pg/ml (IL-1β) vs. 332±105 SEM pg/ml (vehicle); n = 4; p>0.05). In contrast, 4 hours after IL-1β stimulation PGE_2_ concentration in the supernatant of IL-1β treated cells was strongly elevated (1829±640 SEM pg/ml) while vehicle treatment was without effect (191±81 pg/ml SEM; n = 4; p<0.05 (Fig. 3A).

The non-selective COX inhibitor indomethacin (10 µM) and the selective COX-2 inhibitor parecoxib (10 µM) administered to the supernatant of TGC 15 min prior to IL-1β exposure completely aborted PGE_2_ release after 4 hours (Fig. 3B). Statistical significant difference (p<0.05) was achieved for all groups vs. IL-1β + vehicle (n = 3–4/group). In contrast, sumatriptan (10 µM) did neither affect IL-1β induced COX-2 mRNA synthesis nor PGE_2_ release (data not shown). 5-HT_1B/D_ receptor expression in these cells was detected by RT-PCR. Because selective and non-selective COX-inhibitors block IL-1β induced PGE release we conclude that PGE_2_ release from trigeminal ganglia cells is dependent on COX-2 expression and function.

### IL-1β induces delayed CGRP release in trigeminal ganglia neurons

**Figure 4:**
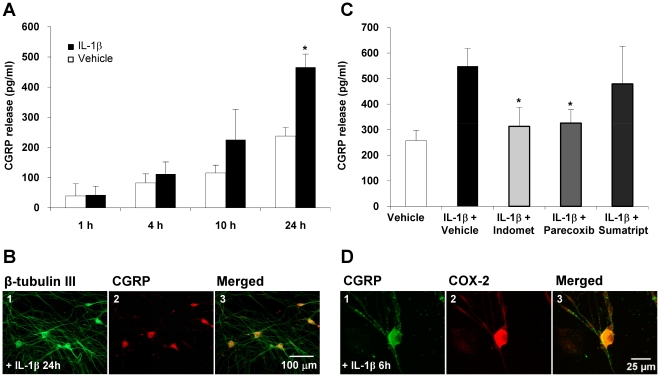
IL-1β induced CGRP release in TGN is dependent on COX-2 activity but not on 5-HT_1B/D_ receptor activation. **A**: IL-1β (10 ng/ml) but not vehicle stimulation for 1, 4, 10 (n = 4) or 24 hours (n = 12) resulted in significantly enhanced CGRP levels in the supernatant of cultured trigeminal ganglia cells at the 24 hrs time point (* p<0.05 vs. vehicle). Earlier time points did not show a significant difference between groups. CGRP release is shown as mean pg/ml ± SEM compared to baseline. Panel **B** shows a representative fluorescent photomicrograph of trigeminal ganglia cells exposed 24 hours to IL-1β (10 ng/ml). Cells were stained with a mouse β-tubulin III antibody, which specifically recognizes neuronal cells (1) and a rabbit CGRP antibody (2). β-tubulin III staining was visualized with an Alexa 488 donkey anti-mouse IgG antibody (green, panel 1). CGRP IgG was recognized by Alexa 594 donkey anti-rabbit antibody (red, panel 2). All CGRP expressing cells stained positive for β-tubulin III (orange, panel 3). **C**: For inhibition experiments TGC were exposed to either parecoxib (10 µM), sumatriptan ( µM), indomethacin (10 µM) or vehicle 45 min prior to 24 h stimulation with IL-1β (10 ng/ml). Incubation with parecoxib (n = 7) and indomethacin (n = 4) blocked CGRP release in the supernatant significantly compared to IL-1β + vehicle (n = 10). Sumatriptan had no effect in the same paradigm (n = 4). CGRP release is shown as mean pg/ml ± SEM compared to baseline. * p<0.05 compared to IL-1β + vehicle. **D**: Immunofluorescence staining shows that CGRP is co-expressed with COX-2 in trigeminal ganglia neurons (n = 3). Cultured trigeminal ganglia cells were exposed to IL-1β (10 ng/ml) for 6 hrs. Cells were stained with an Alexa Fluor 488 donkey anti-mouse IgG labeled rabbit anti-CGRP serum (green, panel 1) and with a rabbit anti-COX-2 antibody, which was recognized by an Alexa Fluor 594 labeled secondary donkey anti-rabbit antibody (red, panel 2). Double stained cells appear orange (panel 3). All CGRP synthesizing cells also stained positive for COX-2. Additionally COX-2 was expressed in glial cells not staining positive for CGRP.

TGN release CGRP upon stimulation with e.g. potassium chloride, a cocktail of inflammatory agents, capsaicin (Durham, 1999) or the cytokine TNFα (Bowen, 2006). To assess whether IL-1β activates trigeminal ganglia neurons (TGN) to release CGRP we studied CGRP concentrations in the supernatant. 24 hours after stimulation CGRP levels were significantly increased compared to vehicle (466±44 SEM pg/ml IL-1β vs. 238±29 SEM pg/ml vehicle; p<0.05 n = 12). CGRP was not different 1, 4 and 10 hours after stimulation with IL-1β (10 ng/ml) compared to vehicle, but a trend towards enhanced CGRP release was seen after 10 hours (226±101 SEM pg/ml (IL-1β) vs. 115±26 SEM pg/ml (vehicle); p>0.05; n = 4) (Fig. 4A).

By using quantitative real-time PCR we further assessed whether the stimulation with 10ng/ml IL-1β also leads to increased transcription of CGRP. We could not observe any differences of CGRP mRNA content between vehicle and IL-1β treated cultures after 1 h, 4 h, 10 h or 24 h (p>0.05; n = 4; data not shown).

To show that CGRP is derived from neurons we co-stained the cultures with CGRP- and β-tub III antibodies 24 hours after exposure to IL-1β or vehicle. All CGRP stained cells also stained positive for β-tub III, clearly indicating that CGRP is expressed by neuronal cells. CGRP could be detected in the cell body and neuronal processes (Fig. 4B). As seen previously in other trigeminal ganglia cell culture studies [19], [32] almost all neurons stained positive for CGRP, whereas *in vivo* in rat and human only approximately 23-50% of all trigeminal ganglia neurons contain CGRP [33], [34]. All neurons staining positive for CGRP were also positive for COX-2, whereas glial cells did only stain positive for COX-2 (Fig. 4C).

### IL-1β induced CGRP release in in trigeminal ganglia neurons is COX-2 dependent

To analyze whether IL-1β induced CGRP release is affected by COX-2 activity, trigeminal ganglia cells were incubated 45 min prior to stimulation with IL-1β with 10 µM indomethacin or 10 µm parecoxib. Both COX inhibitors blocked enhanced CGRP release after 24 hours (548±70 SEM pg/ml for IL-1β + vehicle (n = 10) vs. 326±52 SEM pg/ml for IL-1β + parecoxib (n = 7; p<0.05) and 313±75 SEM pg/ml for IL-1β + indomethacin (n = 4; p<0.05)). There were no significant differences between CGRP release in the IL-1β + COX-inhibitor groups and vehicle + vehicle stimulation (257±40 SEM pg/ml; n = 10; p>0.1). Incubation with Sumatriptan 10 µM did not block IL-1β induced CGRP release (479±147 SEM pg/ml; p>0.1; n = 4) (Fig. 4C). Incubation with a higher dose of sumatriptan (100 µM) did also not change CGRP release in preliminary experiments.

To determine if the same population of trigeminal ganglia neurons expresses CGRP and COX-2, we co-stained trigeminal IL-1β treated (6 hours) trigeminal ganglia cell cultures with CGRP- and COX-2-antibodies. Immunohistochemistry revealed that all neuronal cells expressing COX-2 also stained positive for CGRP, whereas glial cells only stained positive for COX-2 (Fig. 4D).

### PGE_2_ stimulation causes CGRP release in in trigeminal ganglia neurons

**Figure 5:**
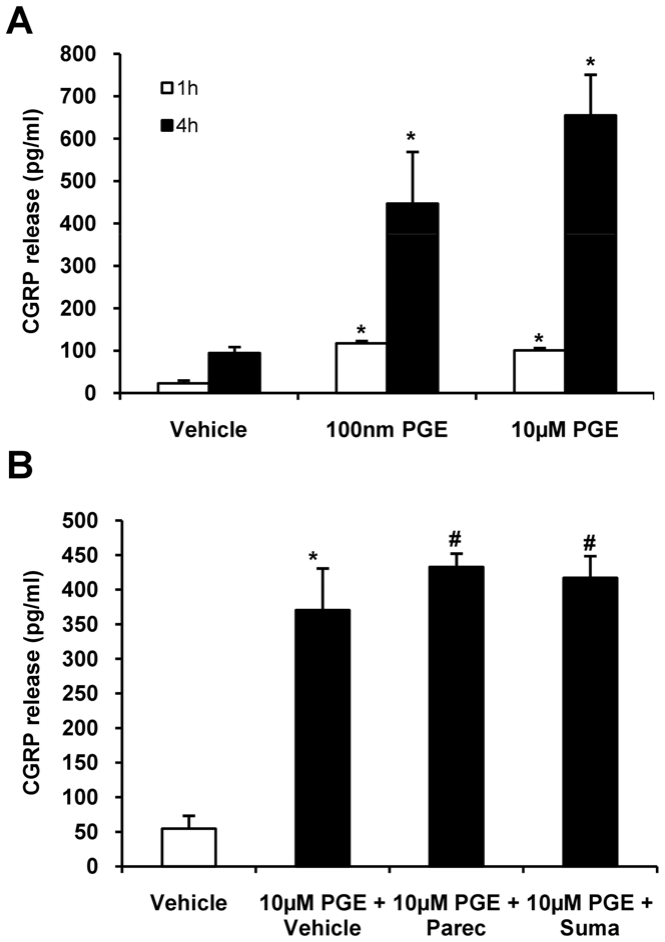
CGRP release in the supernatant of cultured trigeminal ganglia cells after stimulation with PGE_2_. **A**: PGE_2_ stimulation (100 nM and 10 µM) increased CGRP levels within the supernatant time and dose dependently after 1 hour (n = 5/group) and more pronounced after 4 hours compared to vehicle (n = 7/group). **B**: Incubation with 10 µM parecoxib and sumatriptan (10 µM and 100 µM [not shown]) did not alter PGE_2_ release compared to incubation with vehicle (n = 3-4). CGRP is shown as mean pg/ml ± SEM compared to baseline. * p<0.05 compared to vehicle. # p>0.1 compared to PGE + vehicle.

We could show before that IL-1β induces PGE_2_ release in TGC and activates TGN to release CGRP. To determine whether PGE_2_ directly activates TGN as determined by CGRP release, cultured trigeminal ganglia cells were stimulated for 1 hour and 4 hours using two different PGE_2_ concentrations (100 nm and 10 µm). One hour after stimulation with 10 µm PGE_2_ the CGRP concentration in the supernatant was significantly increased (101±32 SEM pg/ml for PGE_2_ vs. 23±8 SEM pg/ml for vehicle; p<0.05; n = 5) and further increased after 4 hours (655±95 SEM pg/ml for PGE_2_ vs. 94±14 SEM pg/ml for vehicle; p<0.05; n = 7). A lower dose of PGE_2_ (100 nM) led to a minor but still significantly increased CGRP concentration after 1 hour (117±45 SEM pg/ml; p<0.05 vs. vehicle; n = 6) and after 4 hours (447±122 SEM pg/ml; p<0.05; n = 5) (Fig. 5A).

There was no effect of the COX inhibitors on PGE_2_ function in this paradigm. We added 10 µm parecoxib (45 min) prior to stimulation with PGE_2_ to the cultures, which did not alter CGRP release after 1 hour and 4 hours compared to vehicle (n = 3–4; P>0.1). There was also no effect on CGRP release by pre-stimulation with sumatriptan 10 µM (Fig. 5B) and 100 µM (Data not shown).

## Discussion

We have shown that cultured primary trigeminal ganglia neurons and glial cells express COX-2 upon stimulation with IL-1β in a dose and time dependent manner leading to PGE_2_ synthesis and protracted CGRP release in trigeminal ganglia neurons. IL-1β induced PGE_2_ and CGRP release was blocked by selective and non-selective COX-2 inhibitors thereby demonstrating dependency on COX-2 enzyme activity. Neither PGE_2_ nor CGRP release were mediated by activation of 5-HT_1B/D_ receptors as the pre-incubation with sumatriptan was without effect.

The trigeminal ganglion (TG) is known to play a key role in the pathophysiology of migraine and other primary headaches. Cultured sensory neurons derived from trigeminal or dorsal root ganglia have been shown to express the characteristics of “differentiated pain sensory” cells [28]. Cultured TG cells have been used to illustrate molecular mechanisms related to migraine pathophysiology e.g. 5-HT_1B/D_ controlled CGRP release [19]–[21], [35], [36].

For stimulation we used IL-1β, a pro-inflammatory cytokine of significant importance in models of inflammatory pain [2], [27], [37] and sensitization [38]. IL-1β has been shown to activate nociceptors to induce pain hypersensitivity in a rat skin nerve preparation [38]. In inflammatory pain IL-1β was identified as the key mediator to induce COX-2 in spinal cord [27] and in cultured dorsal root ganglia cells [39]–[42]. The observation that IL-1β is elevated in plasma during a migraine attack points to an involvement in migraine pathophysiology [5], [6]. High levels of chemokines could stimulate the activation of trigeminal nerves and the release of vasoactive peptides, resulting in inflammation [43].

### IL-1β induced COX-2 and PGE_2_ synthesis resulting in CGRP release

Stimulation of trigeminal ganglia cells with IL-1β led to a strong induction of COX-2 mRNA and protein with subsequently PGE_2_ synthesis. Direct stimulation of TG cells with PGE_2_ resulted in enhanced CGRP release in trigeminal ganglia neurons after one hour and more pronounced after 4 hours. In an *in vitro* preparation of the rat skull fluid-filled cavities electrical and chemical activation of trigeminal afferents resulted in enhanced release of PGE_2_ and CGRP from rat dura mater encephali [44] In a rat trigeminal ganglion cell culture model Jenkins and co-workers identified the EP-2 receptor as the key signaling mechanism for PGE_2_ induced CGRP release [45]. Enhancement of capsaicin induced CGRP release by PGE_2_ has been observed in slices of the trigeminal nucleus caudalis, indicating a prostaglandin induced neuronal sensitization [46].

While these observations may support a role of PGE_2_ in neurogenic inflammation, it is uncertain which COX isoform accounts for the PGE_2_ release. Since COX-1 is constitutively expressed in many cells types (e.g. dural macrophages, fibroblasts) and PGE_2_ release occurs immediately after electrical and chemical meningeal stimulation [44] COX-1 may account for this response. In our model, immediate PGE_2_ release did not occur after stimulation with IL-1β. Additionally, COX-1 mRNA remained unchanged 3 hours after stimulation with IL-1β. In contrast, IL-1β induced COX-2 mRNA expression after 3 hours and PGE_2_ release after 4 hours. PGE_2_ release could be blocked by the selective COX-2 inhibitor parecoxib. These findings provide evidence for a COX-2 mediated pathway.

### Glia-neuron interaction

We found neuronal and glial cells as a source of COX-2 as demonstrated by immunohistochemistry. In particular, a strong stimulus dependent induction of COX-2 by IL-1β was seen in large glial cells. Stimulation of cultured trigeminal cells with PGE_2_ and IL-1β led to CGRP release exclusively in trigeminal ganglia neurons (cell body and neuronal processes). Immunohistochemistry did not reveal any CGRP expression in glial cells which is in line with the findings of others in rat and human trigeminal ganglia [34].

Our findings support a glia-neuron interaction within the trigeminal ganglion. We hypothesize that IL-1β activates glial cells and neurons in the trigeminal ganglion, which leads to the expression of COX-2 in these cells. In turn the COX-2 reaction product PGE_2_ activates trigeminal neurons to release CGRP.

Glia-neuron interaction plays an important role for the normal function of the brain as well in the pathophysiology of many CNS diseases [47]. Over the last years the importance of CNS glia for neuronal function in pain processing has been demonstrated in various experimental pain states [48]. The physiological function of the glial cells within the trigeminal ganglion is not well understood. In the trigeminal ganglia cell bodies of neurons are surrounded by satellite glial cells that can modulate their function and enhance their excitability [49]. In a recently published work IL-1β induced COX-2 expression and PGE_2_ synthesis in cultured trigeminal satellite cells. Stimulation of TGN with conditioned media from these activated satellite cells led to sensitization of TGN resulting in increased CGRP release after stimulation with capsaicin [50]. Activation of satellite glial cells in the trigeminal ganglion modulates the excitability of TG neurons via IL-1β following inflammation associated with hyperalgesia in rats [51]. The involvement of neuron-glia signaling via gap junctions and release of nitric oxide and pro-inflammatory cytokines from the trigeminal ganglia satellite glial cells has been demonstrated recently in experimental models related to migraine pathophysiology [52]–[55]. Interestingly, CGRP receptors are expressed on trigeminal neurons and glial cells. CGRP released by trigeminal ganglia neurons has been shown to function in a paracrine manner to activate trigeminal satellite glial cells to release various cytokines including IL-1β and nitric oxide, a molecule known to be involved in migraine pathophysiology [52], [54]. Additionally CGRP possesses autocrine signaling function properties to increase mRNA levels of CGRP in cultured trigeminal neurons [35]. Our finding that induction of COX-2 expression by IL-1β in trigeminal glial and neuronal cells leads to direct induction of CGRP release from trigeminal neurons supports the notion of an important cross talk between neurons and glial cells in the trigeminal ganglion in processes involved in trigeminally mediated headaches.

### IL-1β induced CGRP release in trigeminal ganglia neurons

In our model exposure of trigeminal ganglia cell culture to IL-1β led directly to delayed CGRP release from TGN 24 hours after stimulation with an earlier non-significant trend after 10 hours. This latency was not expected as IL-1β induced PGE_2_ release was significant after 4 hours and PGE_2_ caused CGRP release after 1 hour. Delayed (24 hours) IL-1β induced CGRP release was observed previously in dorsal root ganglia neurons. Blocking experiments demonstrated that IL-1β might activate proteinkinase C that in turn initiates c-Jun-N-terminal kinase mitogen activated protein kinase followed by activation of nuclear factor-kappaB, which finally induces alpha-CGRP gene expression and neuropeptide release from these sensory neurons [56]. In our hands IL-1β did not lead to an induction of CGRP mRNA synthesis in cultured trigeminal neurons as demonstrated by quantitative RT-PCR. We speculate that a certain threshold of PGE_2_ concentration in the supernatant is necessary to induce gradual CGRP expression, which may take several hours to archive. Direct stimulation of TG with 10 µm PGE_2_ did also not lead to the induction of CGRP gene expression. In line, in another rat model of isolated trigeminal ganglion the infusion of the NO donor glyceroltrinitrate induced the release of CGRP but did not change mRNA levels of CGRP [57]. Therefore, elevated CGRP release in the trigeminal ganglion in our and other models seems to be dependent on enhanced secretion rather than synthesis due to gene expression.

Non-selective (indomethacin) and selective (parecoxib) COX-2 inhibitors aborted L-1β induced CGRP release. In contrast, neither IL-1β nor PGE_2_ induced CGRP release could be blocked by sumatriptan, indicating a release mechanism independent of 5-HT_1B/D_ receptor activation. A COX-2 dependent CGRP release could also be demonstrated in the dura mater in an isolated preparation of fluid-filled rat skull cavities. The COX-2 inhibitor S-flurbiprofen inhibited inflammatory mediator (bradykinin, histamine and serotonin) induced CGRP and PGE_2_ release while the 5-HT_1B/D_ receptor agonist naratriptan was without effect [58]. Our findings demonstrate a direct link between COX-2 activity and CGRP release in TGN. In a slightly different cell culture model of trigeminal ganglia cells potassium chloride and inflammatory cocktail induced rapid CGRP release that could be blocked by sumatriptan [19]. The results of our study point to an alternative pathway of cytokine induced CGRP release in TGN regulated by COX-2 and not affected by sumatriptan.

### Conclusion

**Figure 6:**
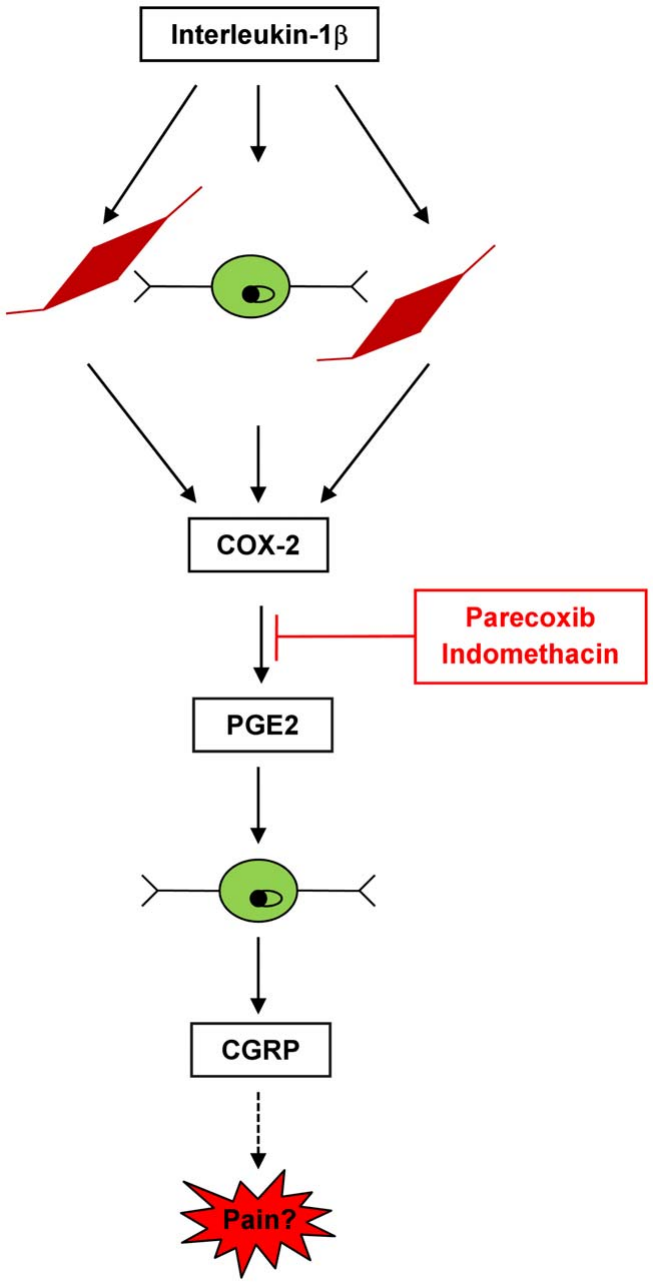
COX-2 dependent induction of CGRP release in trigeminal ganglia neurons. Stimulation with IL-1β leads to the synthesis of COX-2 in trigeminal neurons and glial cells followed by PGE_2_ release. PGE_2_ in turn activatesTGN to release CGRP. PGE_2_ and CGRP release can be blocked by selective (parecoxib) or non-selective (indomethacin) COX-2 inhibitors. The attenuation of CGRP and PGE_2_ release could contribute to the effect of COX-inhibitors to revoke sensitization and to abort pain.

In summary, our results demonstrate that primary trigeminal ganglia cells are able to synthesize COX-2 and PGE_2_ upon stimulation with the cytokine IL-1β in a functionally significant mode resulting in delayed CGRP release in trigeminal ganglia neurons. CGRP release is mediated by a COX-2 dependent pathway and independent of 5-HT_1B/D_ receptor activation (Fig. 6).

COX-2 expression in trigeminal ganglion cells could contribute to the development of pain in trigeminal mediated headaches. Non-selective [23] and selective COX-2 inhibitors [24], [25] abort migraine attacks. The demonstrated attenuation of CGRP release by COX-2 inhibition could at least in part explain the mechanism of COX inhibitors in migraine therapy.
